# HPV-related oropharyngeal cancer early detection in gay and bisexual men is an “orphan” practice: A qualitative analysis among healthcare providers

**DOI:** 10.3389/fpubh.2023.1165107

**Published:** 2023-04-20

**Authors:** I. Niles Zoschke, Sarah L. Bennis, J. Michael Wilkerson, Cynthia L. Stull, Alan G. Nyitray, Samir S. Khariwala, C. Mark Nichols, B. R. Simon Rosser, Charlene Flash, Michael W. Ross

**Affiliations:** ^1^School of Public Health, University of Texas Health Science Center at Houston, Houston, TX, United States; ^2^Division of Epidemiology and Community Health, School of Public Health, University of Minnesota, Minneapolis, MN, United States; ^3^Department of Primary Dental Care, Division of Dental Hygiene, School of Dentistry, University of Minnesota, Minneapolis, MN, United States; ^4^Cancer Center, Medical College of Wisconsin, Milwaukee, WI, United States; ^5^Center for AIDS Intervention Research, Medical College of Wisconsin, Milwaukee, WI, United States; ^6^Department of Otolaryngology-Head and Neck Surgery, University of Minnesota, Minneapolis, MN, United States; ^7^Avenue 360 Health and Wellness, Houston, TX, United States; ^8^Baylor College of Medicine, Division of Infectious Disease, Houston, TX, United States; ^9^Tilman J. Fertitta Family College of Medicine at the University of Houston, Houston, TX, United States; ^10^Department of Family Medicine, School of Medicine, University of Minnesota, Minneapolis, MN, United States

**Keywords:** HPV, oropharyngeal, cancer, detection, gay, bisexual, men, qualitative analysis

## Abstract

**Introduction:**

Among US men, oropharyngeal cancer (cancer of the back of the mouth and throat) is the 8th most common cancer. If detected early, human papillomavirus (HPV)-16-associated oropharyngeal cancer has a high 5-year survival rate. Risk factors such as high numbers of oral sex partners, disparities in smoking and drinking, and low rates of HPV vaccination may put gay and bisexual men at even higher risk for oropharyngeal cancer.

**Methods:**

We recruited 21 healthcare providers in Minneapolis-St. Paul, Minnesota and Houston, Texas to participate in semi-structured interviews. Nurses, physician assistants, dental hygienists, and dentists were asked about their clinical experiences serving gay and bisexual men and opinions on potential interventions for the early detection of oropharyngeal cancer.

**Results:**

Providers typically did not tailor health screenings and examinations for gay and bisexual men. Participants lacked confidence in their ability to effectively implement routine screening for oropharyngeal cancer. The extent to which oropharyngeal cancer screening was incorporated into clinical practice varied by specialty, and practices necessary to detect it were scattered across clinical environments. HIV- and LGBTQ-focused healthcare providers were more aware of HPV-associated oropharyngeal cancer in gay and bisexual men, and appeared readier to act and lead on this issue.

**Discussion:**

Further studies should (1) evaluate protocols for oropharyngeal cancer detection; (2) identify and assess the acceptability of screening in the community; and (3) study how to best close gaps in health services for gay and bisexual men which might contribute to low early detection rates of oropharyngeal cancer.

## 1. Introduction

Oropharyngeal cancer (OPCa) is the 8th most common cancer in men in the US, with one of the highest increases in rates of any cancer (1). The oropharynx includes the back one-third of the tongue, the soft palate and uvula, tonsillar pillars and tonsils, and oropharyngeal walls. Much of this at least partially is visible on an oral inspection (2). The Human Papillomavirus (HPV) is responsible for most cases of OPCa, outpacing carcinogen-induced OPCa (3). Among all HPV-associated cancers, the greatest number of annual cases is attributed to oropharyngeal cancer (3). As of 2018, the number of cases per year of HPV-associated OPCa among men (*N* = 10,600) surpassed the number of cases per year of HPV-associated cervical cancer among women (*N* = 8,100) (4). In the US, OPCa is highly gender-linked; between 2001 and 2017, among men, OPCa incidence increased 2.7% per year (95% CI, 2.5% to 2.9%) to a total of 8.9 cases per 100,000, while no change in incidence occurred among women (a total of around 1.6 cases per 100,000) (5). The rise in OPCa cases is accounted for by HPV-associated OPCa. Among OPCa patients, 91.1% had human papillomavirus (HPV)-16 positive tumors compared to 3.3% of oral cavity (lips, gums, teeth, hard palate, floor of mouth, cheeks, and front of the tongue) cancer cases (6).

Classic OPCa occurs in heavy smokers and alcohol users, while HPV-associated OPCa is associated with oral sexual risk behaviors. HPV-associated OPCa is now recognized as a separate entity with a well-defined risk population: young adult men who do not smoke or drink but engage in high-risk sexual behavior (7). Oral HPV infection risk increases with the number of recent oral sex partners, and data suggests that high rates of oral HPV infection lead to increased rates of OPCa (8). In the US, oral HPV-16 prevalence was six times higher in men (1.8%) than in women (0.3%) (9). In men who reported having two or more same-gender oral sex partners in their lifetime, high-risk oral HPV infection (including HPV 16, 18, and other cancer-causing types), prevalence was 22.2%, compared to 6.8% of men with no lifetime same-sex oral sex partners (*P* = 0.038), representing an enormous health disparity for gay and bisexual men (GBM) (9).

Patients with HPV-positive oropharyngeal cancer have significantly superior survival over those with non HPV-positive oropharyngeal cancer, with half the risk of death (10). In the US, 65% of people with HPV-associated OPCa tumors survived 5 years compared to only 28% of patients with OPCa tumors not associated with HPV (*p* < 0.0001) (11). Because detecting HPV-associated OPCa before it has metastasized leads to reduction in morbidity and mortality, early detection and treatment can have a major impact on psychosocial distress and reduced quality of life from cancer diagnosis (2). Therefore, HPV-associated OPCa is ripe for screening, especially considering its rising incidence.

Early vaccination against carcinogenic HPV (16 and 18) types is critical, too. The Centers for Disease Control and Prevention routinely recommends HPV vaccination for people of all genders, including GBM, from ages 9 to 26, as well as for those who did not receive the vaccine, up to the age of 45 (12). Recent incidence models project vaccine-associated reductions in OPCa will not be realized until 2060, considering current vaccination rates (13). Further, the shift in the burden of OPCa from a younger cohort (35–54 years of age) to an older vaccine-ineligible cohort (65–84 years of age) will continue, due to age and birth cohort effects with a 50% increase in incidence among those 70 years of age and older between 2018 and 2045 (13, 14). Notably, HPV vaccination rates among GBM are below Healthy People 2030 targets (80% adolescents aged 13–15 years) to minimize excess healthcare costs [Increase the Proportion of Adolescents Who Get Recommended Doses of the HPV Vaccine — IID-08 - Healthy People 2030 | health.gov^1^; (15)]. A recent meta-analysis including 78 studies conducted mostly in the US demonstrated that HPV vaccine completion among GBM was 47%, leaving much room to improve (15). This study also demonstrated that GBM under 25 years of age and over 40 were less likely to initiate vaccination than other age categories (15). These data suggest that there may be a window, which narrows with age, for vaccination of GBM to prevent acquisition of carcinogenic HPV types. However, for unvaccinated men or men infected with HPV, the focus must be on early detection, diagnosis, and treatment to improve outcomes.

Currently, there is no approved screening test for HPV-associated OPCa (16). Therefore, it is critical to develop new techniques to identify OPCa early (16). One opportunity may be improving visual inspections, typically involving the inspecting the oropharynx and palpating the lymph nodes in the neck (17) and increasing the frequency of such inspections. Physicians and nurses anecdotally appear less likely to carry out oropharyngeal examinations unless there are specific cancer-associated symptoms. Furthermore, a recent study demonstrated that only about one of four US adults over the age of 30 who get oral healthcare receive oral and oropharyngeal cancer screening (18). In general, there is little information on what healthcare providers are aware of, and practice, with regard to routine examinations to detect OPCa, particularly among GBM. Thus, the purpose of this study was to understand how healthcare workers in two US cities use visual inspection of the oral cavity and specifically the oropharynx in GBM for early detection of OPCa.

## 2. Research design and methods

### 2.1. Methods

We recruited healthcare providers from Houston, Texas and Minneapolis-St. Paul, Minnesota from January to September 2021. Five types of healthcare providers were recruited, including dentists, dental hygienists, physicians, physician assistants, and nurses. In each location, we searched print and social media for healthcare providers that cared for GBM and collected word of mouth recommendations. Clinics, practices, and individual healthcare providers were contacted *via* email, phone, and flyers. After initial contact, interested individuals were directed to complete an eligibility and consent form programmed in Qualtrics©. Eligible and consenting participants then scheduled a 1-h virtual appointment from the study calendar. Participants were not given specific details about the topic prior to beginning the interview but were informed they would be discussing their work with GBM and thoughts on disease screening. Prior to the scheduled interview, participants were emailed a reminder and a link to the virtual appointment.

The interview guide included five questions and several optional probes to explore participants' knowledge of OPCa, care for GBM, screening protocols, education, and opinions toward novel screening methods. See Supplementary material for the full interview guide. All interviews were recorded with participant consent. Participants were compensated for their time and effort with a $100 Amazon gift card. Interview recordings were transcribed then uploaded to Atlas.ti©, a computer-assisted qualitative data analysis software, for analysis.

Saturation was assessed by comparing provider responses across three central interview guide questions. Two coders independently examined provider transcripts to record brief statement summaries and poignant quotes. Finally, the coders compared provider responses for similarities and differences that would indicate saturation. It was determined that the qualitative data was cohering around central themes and that contradicting quotations were sufficiently contextualized for the research team to draw conclusions.

### 2.2. Analysis

We applied a deductive coding approach to generate provisional codes for the final transcripts (19). Two independent coders reviewed transcripts and created a preliminary codebook of code titles, definitions, specific uses, and relevant quotations. The two coders then compared codebooks and merged or expanded codes to generate a final codebook, which was re-applied to all transcripts. A total of 45 codes were developed. The research team then applied thematic analysis to the coded data (20). This process involved examining the coded data, looking for patterns, commonalities, divergences, highlights, special cases, and outcomes that were not anticipated by the literature review (20). These patterns were then closely examined by the research team to determine if the emerging concepts were rooted in the ideas and perspectives of the researchers or embedded in the language and experiences of the study participants, or both. Phenomena that seemed authentically rooted in both the qualitative data and literature review (either as complementary or divergent) emerged as themes.

## 3. Results

### 3.1. Participant demographic characteristics

The demographic characteristics for participants are displayed in Table 1. Participants had an average age of 43.8 years and an average of 18 years in practice. There was a fairly even split between cisgender men and cisgender women participants. A just over a third of participants identified as a sexual minority. While a majority of participants were white, three participants were Asian and five were Hispanic/Latino. Participants worked in a range of healthcare settings.

**Table 1:** Participant demographic characteristics.

**Participant demographics**	**Mean**	**Range**
Age (years)	43.8	28–70
Years in practice	18	4–47
**Gender**	**N**	**%**
Female	12	54.5
Male	9	40.9
Missing	1	4.5
**Sexual minority status**		
Yes	8	36.4
No	14	63.6
**Race**		
White	16	72.7
Asian	3	13.6
Missing	3	13.6
**Ethnicity**		
Hispanic/Latino	5	22.7
Not Hispanic/Latino	16	72.7
Missing	1	4.5
**Practice Information**	**N**	**%**
**Provider type**		
Physician (MD/DO)	4	18.2
Dentist (DDS/DMD)	4	18.2
Nurse (RN/NP/LPN/CNM)	7	31.8
Dental Hygienist (RDH)	4	18.2
Physician Assistant (PA-C)	2	9.1
Other	1	4.5
**Practice type**		
Federally Qualified Health Center	7	31.8
Hospital/Medical Center	1	4.5
Practice Network/HMO	3	13.6
Private Practice	5	22.7
Academic Center/School	2	9.1
Community Health Clinic	1	4.5
Public Health	2	9.1
Other	1	4.5%
**Practice location**		
Minnesota	11	50.0
Texas	10	45.5
Not currently practicing	1	4.5

FQHC, Federally qualified health center.

### 3.2. Supporting themes

Seven themes emerged during analysis: (1) *treating patients the same*, (2) *Healthcare providers of all types are aware of HPV-associated cancers, but can't always address the problem; patients are less aware*, (3) *guidance on OPCa screening and prevention is lacking, especially among GBM*, (4) *lack of funding and time are barriers to comprehensive oral health examinations*, (5) *discomfort with talking about sex can be a barrier to OPCa detection*, (6) *screening for OPCa is an “orphan,”* and (7) *HIV- and Lesbian, Gay, Bisexual, Transgender, and Queer (LGBTQ)- specific clinicians are at the forefront of OPCa screening and prevention*.

#### 3.2.1. Treating patients the same

When participants were asked whether they tailor HPV or OPCa prevention services for GBM patients, most said they did not. For example, one Registered Dental Hygienist working at a Minneapolis-St. Paul private practice said:

“Actually, no [we don't tailor screenings for GBM]. I was thinking about that when you had sent me a little preview on that. I was like, “Do we?” It's interesting because it only comes up organically. We don't have a box that's checked, “what is your interest?” No, I think we do just the same general screenings we do with every patient because... Like oral cancer screenings, those are the kinds of things that would show up any way if there was an association with anything. Not really anything different.”

This quotation illustrates a sentiment shared by many of the participants from all professions that providing uniform services to all patients, ensuring everyone receives equal treatment, was the best practice. Some physicians, physician assistants, and nurses asked more in-depth sexual health history questions if they knew the patient was a GBM. For example, when patients reported to nurses they had oral sex with men, it prompted oral swabs for chlamydia and gonorrhea. Patient HPV vaccination status was a key indicator prompting physicians, physician assistants, and nurses to discuss the risks of HPV and associated cancers with patients. Some participants justified treating all patients the same because they were concerned about making GBM feel singled out, as all patients who are sexually active are at risk for HPV. This fact led one participant to report they believed all patients should receive the same OPCa prevention services.

#### 3.2.2. Healthcare providers of all types are aware of HPV associated cancers, but can't always address the problem; patients are less aware

Healthcare professionals of all types were aware that HPV causes cancer. The majority of participants reported recommending GBM patients apply safer sex practices to prevent HPV. However, among the nurses and physicians interviewed, cervical cancer was more commonly discussed than OPCa. Due to gaps in training and service, opportunities for prevention beyond vaccination were missed. For example, two nurse participants stated they were not equipped to screen for OPCa. Additionally, participants in general reported that their patients were less aware of HPV-associated cancers. For example, one Doctor of Dental Surgery working at a Houston Federally Qualified Health Center (FQHC) said:

“That's something that I have been mentioning more now to patients. We talked a lot about HIV pretty openly and frequently with my patients, and we also talked a lot about other STIs in general. I feel like HPV has been something that has not been talked about. For the most part, a lot of patients don't really know what it is or that it even exists.”

This quotation, combined with the experience of another nurse participant who said their patients never ask about OPCa, demonstrates the level of awareness about HPV and the perception of resultant risk to health among patients is low.

#### 3.2.3. Guidance on OPCa screening and prevention is lacking, especially among GBM

Clearly, dentists and dental hygienists routinely looked inside patients' mouths and throats to screen for diseases. However, physicians, physician assistants, and nurses expressed they may only look inside a patient's mouth if a specific symptom is presented. Participants in general commonly reported that a lack of training, institutional guidance, education, and standard screening practices on OPCa was a barrier to effective prevention. For example, one nurse practitioner at a Minneapolis-St. Paul medical center said, “I need to check into that, what the clinical outcomes are in that arena. Is it really worth our time doing? Is it something that's needed to do? I know the USPSTF [United States Preventive Services Task Force] doesn't have any recommendations for that.” This quotation demonstrates the common experience that the lack of clinical recommendations makes prevention approaches unclear. Additionally, only a few participants reported receiving adequate training to address OPCa concerns unique to GBM.

#### 3.2.4. Lack of funding and time are barriers to comprehensive oral health examinations

Physicians, physician assistants, and nurses reported that HIV and STI prevention and treatment were central to their work because of emphasis from their clinics, funders, and qualifying patient healthcare coverage. Screenings that were not recommended by national institutions were typically not covered in a regular check-up due to time and resource constraints. Generally, participants reported being tight on time with patients, occupied with existing routine screenings. For example, one Minneapolis-St. Paul-based dentist mentioned that between their practices' dentist and dental hygienist, no more than 5 min is spent looking inside the oral cavity for signs of cancer. Another Minneapolis-St. Paul-based dentist stated they often tried to fit an hour's worth of care into a 20-min visit. Additionally, one nurse practitioner working in a Houston public health setting said, “We don't have [the] ability [for full-body screening]. We don't have that ability, funding, or the clinical… [sic] to get that done. Like I said, we were very limited. We're working toward that to do the full screening, but otherwise, we can't do that.” Combined, these experiences demonstrate that time and money are major barriers to detecting OPCa early when it is most critical.

#### 3.2.5. Discomfort with talking about sex can be a barrier to OPCa detection

Participants reported that in some cases, GBM patients had guilt and shame about their sexual identity and sexual practices, which can prevent healthcare utilization and impede discussions on oral health. However, competency and comfort talking about these issues varied across participants' roles and healthcare settings. For example, several physicians, physician assistants, and nurses reported having developed skills to discuss sexual health, particularly with GBM. But one dental hygienist in Minneapolis-St. Paul stated that discussing HPV prevention with youth and their families can be difficult. Another Minneapolis-St. Paul-based nurse participant stated that even though their practice had come a long way in destigmatizing discussions on sex and sexuality with patients, these approaches didn't appear common. This participant said that a number of patients report not being able to talk about same sex behaviors because of histories of trauma and judgment. Lastly, another dental hygienist working in a Minneapolis-St. Paul private practice said, “It is a bit of a tough... It is a weird, tough topic to, like, talk about.” Together these experiences underscore that discomfort with discussions about sex serves as a critical barrier to effective OPCa screening and examination.

#### 3.2.6. Screening for OPCa is an “orphan”

Participants reported that healthcare providers in general were missing opportunities to prevent OPCa. Only a few participants reported being sure their practice had a written protocol for OPCa screening, each working in dentistry in either private practice or a practice network. Only one participant, a physician assistant working in an FQHC, reported having a written protocol for general health screenings for GBM. Participants stated that HPV-associated cancer prevention and detection requires a number of activities that are scattered across general practice, dentistry, and specialized oral healthcare. To illustrate this point, one doctor at a Minneapolis-St. Paul community health clinic said, “So I think [OPCa education and screening is] a little bit of an orphan and I'm guessing that's part of why this research is being done.” This comment represents a prevailing notion among participants that OPCa prevention doesn't have a single clinical home. For example, dentists and dental hygienists sometimes viewed discussions about oral sex as not their purview, yet physician assistants, and nurses lack the training to detect cancers of the mouth and oropharynx. Additionally, the separation between general healthcare and dentistry may create gaps in OPCa prevention. The separation can manifest in several ways; physicians, physician assistants, and doctors, typically refer oral health concerns to dentists. But dentists and dental hygienists don't typically see sexual health prevention as in their scope of work. Furthermore, dentists and dental hygienists lack the specialized equipment to examine deep in the throat and study participants were concerned that OPCa in the throat may go undetected prior to referral to an ear, nose, and throat specialist.

#### 3.2.7. HIV-and LGBTQ-specific clinicians are at the forefront of OPCa screening and prevention

Healthcare providers that work in HIV- and LGBTQ-specific healthcare settings appeared more prepared and readier to bridge the gap between providing sexual health services and OPCa screening. For example, among the few participants who reported receiving adequate training about addressing OPCa among GBM, most identified as sexual minorities themselves. Perhaps lived experience and familiarity with LGBTQ issues cues healthcare providers off to address these concerns. One Houston-based dentist who worked in HIV-centered care suspected that dentists who serve the general population may find candid discussions about GBM sexual behaviors difficult to foster, even though their own practice was very open with patients about these issues. This candor allows for patients to be open and honest about sexual practices and other risk factors for OPCa. The experiences of professionals in HIV- and LGBTQ-specific healthcare settings could be leveraged to improve practice, as well. For example, another Houston Doctor of Dental Surgery working in the same HIV-focused practice referenced earlier said, “[Our HIV-focused training] could easily be expanded to talk about HPV, right? Because we do talk a lot about HPV with HIV. And all of these people [in our training center] are very gay friendly, if not gay themselves. All the dentists here.”

Another nurse practitioner in Houston indicated that they were more aware of HIV-associated oral diseases, and paid close attention to oral lesions because of awareness surrounding GBM sex practices. Additionally, one Minneapolis-St. Paul-based doctor indicated that patients living with HIV may be getting more screening for OPCa due to routine HIV-associated oral health visits. Similarly, one nurse practitioner working in Minneapolis-St. Paul said that young people living with HIV receive aggressive preventive treatment and therefore may have oral health issues like OPCa detected earlier.

## 4. Discussion

### 4.1. Discussion of findings

The key findings of this study are that frontline healthcare providers were not confident in conducting OPCa assessments among GBM, and few medical and dental practices had written protocols for conducting such assessments. Additionally, we found that healthcare providers believe patients don't know that oral HPV infection, and therefore oral sex, are risk factors for OPCa. This finding is consistent with, and extend, what has previously been reported in research on OPCa. Williams et al. (21) found public awareness for OPCa is low and most people are not aware that HPV can cause OPCa. Our study on healthcare provider experiences suggests that gay and bisexual men are similar to other members of the general public in having low awareness of OPCa and knowledge of HPV as a causative agent.

Additionally, we found that healthcare providers view patient stigma associated with being GBM as a barrier to effective OPCa prevention. Several study participants stated that hesitancy to discuss sex and sexuality between healthcare providers and patients may prevent important conversations which may reveal risk for HPV-associated OPCa. This hesitancy goes both ways; in some cases, patients are hesitant to disclose same sex activity. In other cases, healthcare providers, such as dentists and dental hygienists, are uncomfortable asking patients about sex practices. These impressions correspond to literature on the subject. Whitehead et al. (22) found that being closeted to healthcare providers may lead to lower rates of healthcare use among GBM, and Facione and Facione (23) demonstrated that prejudice against LGBTQ people is associated with reduced rates of cancer screening. However, Rindal et al. (24) demonstrated that among a group of 36 dental healthcare providers, 90% reported being highly comfortable with asking patients about sexual behaviors during oral HPV screening, and 69% of patients surveyed (*N* = 1,025) reported being comfortable if asked about them. Perhaps the findings in Rindal et al. (24) indicate that conversations about sexual activity are becoming more commonplace in dentistry.

Appropriate practices to detect HPV-associated OPCa in general are unclear (16). Furthermore, most participants in this study stated they didn't tailor screening services to GBM, which aligns with recommendations that HPV-associated cancer prevention services be promoted to all patients regardless of sexual orientation (25). These conditions may explain why participants lack confidence in approaching OPCa prevention specifically among GBM. However, there is clear evidence that oral HPV infections are more prevalent among GBM (9), and that early detection can prevent excess OPCa morbidity (26). This appears to be a critical concern as GBM already experience cancer disparities (27). Treating all patients equally may fail to equitably address a potential health disparity among GBM which OPCa may represent. Therefore, future epidemiologic studies should investigate whether special attention to detect OPCa early among GBM decreases disparities in OPCa morbidity and mortality.

Some of the results of this study were unexpected and address an important gap in the literature surrounding GBM OPCa disparities. For example, we did not anticipate the central theme of the qualitative outcomes: OPCa early detection and prevention services may be an “orphan” in healthcare, meaning that no single healthcare venue houses all the services necessary to address the problem. Frontline healthcare providers like nurses and physician assistants commonly gather sexual health histories and may tailor assessment questions based on sexual activity to learn more about disease risk such as HPV. However, nurses and physician assistants lack the specialized training to conduct an OPCa examination. These skills usually reside among doctors and dentists. Dentists and dental hygienists said that tailored conversations with GBM patients about their sexual activity was not within their purview, and they may lack training on how to ask these questions. Dentists and dental hygienists have the skills to detect OPCa, but may not pay special attention to GBM who may be at higher risk for HPV-associated OPCa. A study by Stull et al. (28) suggested to improve comfort and confidence of dental providers in having HPV-related conversations, skills-based training and multiple opportunities to practice communication technique are needed. Furthermore, dentists and dental hygienists lack the training and specialized equipment that ear, nose, and throat specialists have available to look for OPCa deeper in the throat. Integrating dental and medical practices, such as conducted in many FQHCs, may improve collaborative OPCa prevention, availing the expertise of each healthcare provider (29).

Healthcare providers who work in HIV- and LGBTQ- specific healthcare settings appear to be strongly positioned to lead in closing critical gaps in OPCa early detection and prevention among GBM. Because these specialized healthcare providers lead destigmatized clinical environments, services are more comprehensive and individualized. This environment may enable healthcare professions to ask about individual risk factors and ultimately to look more closely for oral lesions, which may indicate OPCa. Our findings add to the evidence that dentists and dental hygienists are well positioned to develop protocols for oral HPV detection and that such procedures are feasible and acceptable to both dental healthcare providers and patients (24).

HIV- and LGBTQ- specific healthcare centers represent an important opportunity for increasing healthcare system capacity for OPCa early detection and prevention. In a similar context, dozens of interventions have aimed to integrate cervical cancer screening into HIV treatment services, and while outcomes data are scarcely reported, such interventions are deemed acceptable and feasible among women living with HIV (30). Furthermore, researchers who conducted a qualitative study on social support among GBM living with prostate cancer recommend healthcare providers account for the unique support networks GBM have when referring them for support for their diagnosis (31). It is likely that clinicians who specialize in providing healthcare to LGBTQ patients can integrate new OPCa prevention activities into their workflows, assist GBM to identify tailored cancer survivor networks, and make key recommendations on how to institute such processes broadly across health and dental care.

### 4.2. Limitations

Findings from this analysis should be understood given three limitations. The study sample was drawn from only two cities in the US potentially limiting transferability to other regions. Additionally, because we interviewed physicians, physician assistants, nurses, dentists and dental hygienists, our findings may not represent healthcare providers in other categories. Lastly, because we interviewed a total of 22 participants among five separate professions, examining differences between specific professions was challenging. Despite these limitations, this analysis highlights gaps in OPCa early detection and prevention and identifies opportunities to leverage the experiences of HIV- and LGBTQ- specific healthcare providers to improve OPCa screening.

### 4.3. Conclusion

Methods to best prevent OPCa among GBM are not well understood. Findings from this analysis highlight an opportunity to train primary care and dental providers on how to identify GBM in their practice and to screen these men for abnormalities indicative of possible OPCa. Healthcare providers who specialize in serving people living with HIV or who identify as GBM are well positioned to lead practice-based recommendations and design training for how to systematically detect HPV-associated OPCa. Further studies should (1) better characterize rates of OPCa among GBM; (2) evaluate protocols for OPCa detection; (3) identify and assess the acceptability of screening in the community; and (4) study how to best close gaps in health services for GBM which might contribute to low early detection rates of OPCa. Informed by findings from this analysis and additional research, screening guidelines should be formalized and incorporated into routine preventative care.

## Data availability statement

The datasets presented in this article are not readily available because it may contain identifying details even if redacted. Participants were assured we would not share the data beyond the interviewers and PI. Requests to access the datasets should be directed to mwross@umn.edu.

## Ethics statement

The studies involving human participants were reviewed and approved by University of Minnesota Human Research Protection Program, UTHealth Committee for the Protection of Human Subjects. The patients/participants provided their written informed consent to participate in this study.

## Author contributions

IZ: first authorship, primary data collection, data analysis, and primary authorship. SB: equal contribution, data collection, and data analysis. JW and CS: equal contribution and senior authorship, data interpretation, and revision. AN, SK, and CN: equal contribution, data interpretation, and revision. BR: equal contribution and senior authorship, conception and design, data interpretation, and revision. MR: equal contribution and last authorship, conception and design, data interpretation, and revision. All authors contributed to the article and approved the submitted version.

## Footnotes

^1^ https://health.gov/healthypeople/objectives-and-data/browse-objectives/vaccination/increase-proportion-adolescents-who-get-recommended-doses-hpv-vaccine-iid-08
