# Supplementary material for: HPV-related oropharyngeal cancer early detection in gay and bisexual men is an “orphan” practice: A qualitative analysis among healthcare providers
Source: Front Public Health. 2023 Apr 20;11:1165107. doi: 10.3389/fpubh.2023.1165107 (PMC10162013; doi:10.3389/fpubh.2023.1165107)
Supplement: Supplementary file 1 [file Data_Sheet_1.docx]

Supplementary Material

**HPV-related oropharyngeal cancer early detection in gay and bisexual men is an “orphan” practice: A qualitative analysis among healthcare providers**

**I. Niles Zoschke^1^, Sarah L. Bennis^2^, J. Michael Wilkerson^1^, Cynthia L. Stull ^3^, Alan G. Nyitray^4,5^, Samir S. Khariwala^6^, Mark Nichols^7^, B. R. Simon Rosser^2^, Charlene A. Flash^7^, Michael W. Ross^8^**

^1^School of Public Health, University of Texas Health Science Center at Houston, Houston, TX, United States

^2^Division of Epidemiology and Community Health, School of Public Health, University of Minnesota, Minneapolis, MN, United States

^3^Department of Primary Dental Care, School of Dentistry, Division of Dental Hygiene, University of Minnesota, Minneapolis, MN, United States

^4^Cancer Center, Medical College of Wisconsin, Milwaukee, WI, United States

^5^Center for AIDS Intervention Research, Medical College of Wisconsin, Milwaukee, WI, United States

^6^Department of Otolaryngology-Head and Neck Surgery, University of Minnesota, Minneapolis, MN, United States

^7^Avenue 360 Health and Wellness, Houston TX, United States

^8^Baylor College of Medicine, Division of Infectious Disease, Houston, TX, United States

^9^Tilman J. Fertitta Family College of Medicine at the University of Houston, Houston, TX, United States

^10^Department of Family Medicine, School of Medicine, University of Minnesota, Minneapolis, MN, United States

*** Correspondence:**Corresponding author: Michael W. Ross, mwross@umn.edu

# Supplementary Data

Interview Guide for Healthcare Providers on Experiences Treating Gay and Bisexual Men

**Q1. We are interested in your experiences treating gay, bisexual and other men who have sex with men. If a patient discloses they are a gay or bi man, do you conduct different screening practices for them compared to your heterosexual patients that are men?**

***Probes:***

- 1. Could you give examples of health screenings you conduct?
  2. Could you talk about which health screenings have evolved the most since you began practicing?

**Q2. In this study, we are particularly interested in how you screen for oropharyngeal cancer (OPCa). Would you tell me how you screen patients for OPCa during an appointment?**

***Probes:***

- 1. How is the patient screened (lymph nodes, visual inspection of the mouth and oropharynx, etc.?)
     1. How is screening different if the patient is a GBM?
        1. *If provider does not screen, change language to hypothetical situations (how would screening be different etc.)*
  2. How do you decide who to screen? (presenting with symptoms, disclose certain sexual behaviors)
  3. Do you have a written protocol that you follow?
     1. Would you share the protocol with us and talk about the benefits and challenges with the protocol?

**Q3. Think about a typical GBM patient with whom do you do an OPCa screening. Would you describe a typical case and walk me through your interactions with the client at risk of OPCa?**

***Probes:***

- 1. How is the topic introduced to the patient, if at all?
  2. How much time do you dedicate to OPCa screening during an appointment?
  3. Are there times you prioritize other things in an appointment over OPCa screening? (running late, patient has specific health concern)
  4. How are screening results documented (open clinical notes or a particular field in the EMR with a standardized template or a tailored template)?
  5. What do you do if you see something unusual that might raise concerns about the possibility of OPCa?
  6. Do you ever take a photo or video of the abnormality (e.g., as part of a referral)?
     1. Can you talk me through what you photograph and your experience with taking photos?
  7. How do you explain to the patient with an unusual finding what will be the next steps?
     1. What educational materials do you provide (*request a copy*)?
  8. What is the care plan, including linkage to other services and follow-up appointments?
  9. What protocols do you have for ensuring a patient shows up to referral appointments and how do you get the results of that visit?
  10. What do you do differently during follow-up appointments?
      1. How is it different for a patient who is at risk vs. a patient diagnosed with OPCa?
      2. How does the frequency of OPCa screening change for a patient before and after OPCa concerns or diagnosis?
      3. How is the frequency of OPCa screening different for your GBM patients?
  11. In your experience, where are patients most often lost to follow-up?
  12. How do you re-engage patients at high risk for OPCa who are lost to follow-up?
  13. Who should conduct OPCa screening?

*Now, we would like to learn about training you’ve received on OPCa.*

**Q4. Reflecting on the training you’ve had about OPCa, could you talk about training and education you’ve received on oropharyngeal cancer?**

***Probes:***

- 1. What was useful?
  2. What else would you have liked to learn about?
  3. Who provided the trainings (university, mentor, CME provider)?
  4. What aspects of your training was about OPCa among GBM?

**Q5. One of the goals of this study is to look at the feasibility and acceptability of mouth selfies that could be sent to trained providers that screen for OPCa. I’m interested in what you think about this idea?**

***Probes:***

1. What interests you most about this approach? (save time, screen more patients)
2. What concerns do you have about this method of screening? (quality of photos, insurance, security)
3. Who should receive and examine these pictures?
4. What about the idea of taking photos of the oropharynx in the same way as dentists take x-rays at regular intervals. How helpful would this be in practice?
5. How would you securely communicate with patients about these images?
6. Do you think your GBM patients would be interested in this screening method?
7. How could this intervention be made more acceptable to GBM patients?
8. How could this intervention be made more accessible to all GBM?

**
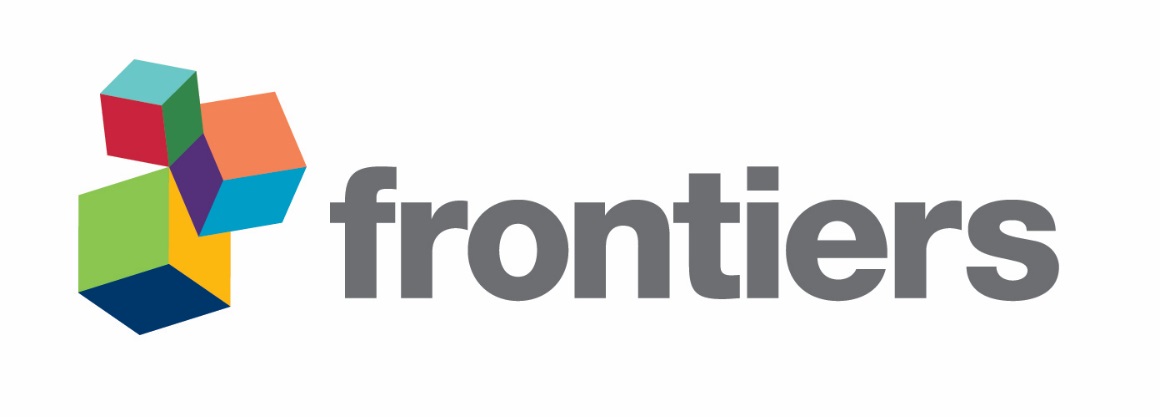
**
